# Antibody to Arenaviruses in Rodents, Caribbean Colombia

**DOI:** 10.3201/eid1707.101961

**Published:** 2011-07

**Authors:** Salim Mattar, Camilo Guzmán, Justiniano Arrazola, Ella Soto, José Barrios, Noemí Pini, Silvana Levis, Jorge Salazar-Bravo, James N. Mills

**Affiliations:** Author affiliations: Universidad de Córdoba, Montería, Colombia (S. Mattar, C. Guzmán, J. Arrazola, E. Soto, J. Barrios);; Instituto Nacional de Enfermedades Virales Humanas “Dr. Julio I Maiztegui,” Pergamino, Argentina (N. Pini, S. Levis);; Texas Tech University, Lubbock, Texas, USA (J. Salazar-Bravo);; Centers for Disease Control and Prevention, Atlanta, Georgia, USA (J. Mills)

**Keywords:** arenavirus, rice rats, common cane mouse, sigmodontine rodents, viruses, Caribbean Colombia, letter

**To the Editor:** The ≈20 recognized arenaviruses in the Americas are hosted by rodents of the family *Cricetidae*; 1 exception may be hosted by a bat (genus *Artibeus*, family *Phyllostomidae*) (*1*). Pichindé virus, hosted by O*ryzomys albigularis*, was described from animals in the Pichindé Valley near Cali, Colombia (*2*), and antibody reactive to Pichindé virus was found in 2 of 82 serum samples from humans in the same area. No studies of arenavirus infection in rodents or humans have been conducted in Colombia since 1971. Although Pichindé virus is not associated with human disease, Guanarito virus, which is hosted by *Zygodontomys brevicauda*, the short-tailed cane mouse (*3*,*4*), causes Venezuelan hemorrhagic fever in the Venezuelan state of Portuguesa (*5*). This state borders on Colombia, and *Z. brevicauda* is a common species in Caribbean Colombia. Our aim was to determine the prevalence of antibody to arenaviruses among wild rodents in this region.

During November 1, 2008–June 10, 2009, we trapped 322 rodents in 3 rural localities in the Department of Córdoba, Colombia (Monteria, Vereda El Escondido, 8°34.183′N, 75°42.776′W; Sahagun, Vereda Las Llanadas, 8°56.533′N, 75°20.909′W; and Lorica, Colegio Instituto Técnico Agrícola, 9°24.067′N, 075°75.707′W). The landscape at the study sites is dominated by tropical savanna, small patches of forest, and cultivated land. Live-capture traps were set in a variety of habitats at each locality, and captured rodents were processed according to methods by Mills et al. (*6*). Rodents were anesthetized, and blood (by cardiac puncture) and tissue (liver, lung, spleen, hearty, kidney) samples were collected into individual cryovials, placed in liquid nitrogen in the field, and transferred to freezers and stored at –80°C at the Instituto de Investigaciones Biológicas del Trópico, Universidad de Córdoba, Montería, Colombia. Rodent species were identified on the basis of morphologic analyses of formalin-fixed carcasses; chromosomal data and mitochondrial DNA sequencing of the cytochrome b gene were used to confirm identification of most antibody-positive animals. Alcohol-preserved voucher specimens are archived at the Museum of Texas Tech University (Lubbock, TX, USA).

Blood samples were tested for arenavirus immunoglobulin (Ig) G by indirect immunofluorescent antibody assays. Guanarito and Pichindé virus–infected Vero E6 cells were used as antigens on spot slides. The secondary antibody was a fluorescein-conjugated goat, antimouse IgG. Serum samples were screened at a dilution of 1:10 and endpoint titers were measured by using serial 2-fold dilutions (1:10–1:320) (*7*). Attempts to amplify viral RNA in tissues by reverse transcription PCR were unsuccessful.

We collected 210 sigmodontine rodents of 3 species: 181 *Z. brevicauda*, 28 *Oligoryzomys fulvescens*, and 1 *Oecomys concolor*. Eleven serum samples, 10 from *Z. brevicauda* and 1 from *O. fulvescens* rodents, had detectable arenavirus antibody. Three *Z. brevicauda* rodent samples had antibody reactive to both Pichindé and Guanarito virus, and 7 more were positive for either Pichindé or Guanarito arenaviruses (Table).

**Table:** Comparison of PICHV and GTOV antibody titers in rodents after serologic screening of 210 sigmodontine rodents, Córdoba, Colombia, November 1, 2008–June 10, 2009*

Specimen no.	Species	PICHV titer	GTOV titer	Rural locality
49	*Zygodontomys brevicauda*	**40**	<10	Monteria
70	*Z. brevicauda*	**80**	10	Monteria
105	*Z. brevicauda*	**40**	<10	Monteria
107	*Oligoryzomys fulvescens*	20	<10	Monteria
272	*Z. brevicauda*	**80**	10	Monteria
289	*Z. brevicauda*	20	<10	Monteria
317	*Z. brevicauda*	**40**	<10	Monteria
344	*Z. brevicauda*	20	<10	Monteria
345	*Z. brevicauda*	<10	20	Monteria
209	*Z. brevicauda*	10	**80**	Lorica
211	*Z. brevicauda*	<10	**40**	Lorica

*PICHV, Pichindé arenavirus; GTOV, Guanarito arenavirus. Titers in **boldface** are >4-fold higher than titers for the other antigen.

We used only 2 viral antigens in our screening belonging to the 2 viruses that are either known to occur in Colombia (Pichindé virus) or known to be hosted by species that we captured (Guanarito virus). Among the 10 *Z. brevicauda* samples with detectable antibody, 5 reacted only to Pichindé virus antigen or their antibody titer to Pichindé virus was at least 4-fold higher than their titer to Guanarito virus (Table), suggesting those rodents were infected with Pichindé or a closely related virus. Additional studies, including isolation and sequencing are needed to definitively identify this virus.

Surprisingly, only 2 *Z. brevicauda* rodents (1.1%) had antibody only to Guanarito virus or had a 4-fold greater titer to Guanarito virus, much lower than the 15% antibody prevalence in the same species in the Venezuelan hemorrhagic fever–endemic area, Portuguesa State, Venezuela (*5*). Our testing protocols differed from the earlier study, and we have not definitively identified Guanarito virus in those 3 rodents; nevertheless, this low prevalence might help explain the absence of Venezuelan hemorrhagic fever in Colombia, although inadequate surveillance is a second possible explanation.

The single antibody-positive *O. fulvescens* rodent had a low antibody titer only to Pichindé virus. This apparent 4% antibody prevalence is based on only 28 mice. The significance of this finding is not clear but may represent spillover or an undescribed arenavirus specific to the species *O. fulvescens*. Again, additional studies are needed.

Our results demonstrate the presence of >1 arenaviruses circulating among common rodent hosts in Caribbean Colombia. We emphasize that many New World arenaviruses are likely cross-reactive to the antigens we used; recovery and sequencing of viral RNA will be essential to fully characterize these viruses. Hemorrhagic fever of arenaviral origin should be included in the differential diagnosis of tropical fevers, at least in our study region. As the human population of the rural Department of Córdoba and adjacent areas of the Caribbean coast of Colombia continues to increase, the potential for arenavirus-related disease could become a public health concern.
